# An *in vitro* and *in silico* evaluation of the antibacterial activity of the bioactive compounds in Majapahit (*Crescentia cujete* L.) fruit

**DOI:** 10.14202/vetworld.2019.1959-1965

**Published:** 2019-12-13

**Authors:** Sri Rahmaningsih, Hernik Pujiastutik

**Affiliations:** 1 Department of Aquaculture, Faculty of Fishery and Marine Sciences, University of PGRI Ronggolawe, Tuban 62381, East Java, Indonesia; 2 Department of Biology Education, Faculty of Teacher Training and Education, University of PGRI Ronggolawe, Tuban 62381, East Java, Indonesia

**Keywords:** antibacterial, *Crescentia cujete*, *in silico*, *in vitro*, Majapahit fruit

## Abstract

**Background and Aim:**

Majapahit (*Crescentia cujete* L.) fruit extract acts as a natural antibacterial agent due to its bioactive constituents such as tannins, flavonoids, triterpenoids, and saponins. The aim of this study was to determine the antibacterial activity of Majapahit fruit against *Vibrio harveyi* both *in vitro* and *in silico*.

**Materials and Methods:**

Column chromatography, minimum inhibitory concentration (MIC) determination, and transmission electron microscopy (TEM) were used for *in vitro* analysis. *In silico* analysis was performed using PubChem^®^ database, Pass Online (Way2Drug.com©), Search Tool 17 Interacting Chemicals (STITCH), and UNIPROT database (https://www.uniprot.org/).

**Results:**

The MIC was found to be 0.313 mg/mL. Within the concentration range of 0.313 mg/mL-10 mg/mL, Majapahit fruit extract could inhibit the growth of *V. harveyi*, while lower concentrations of 0.078 mg/mL and 0.165 mg/mL indicated the presence of bacterial growth. The pathogenic mechanism of *V. harveyi* on vannamei shrimp (*Litopenaeus vannamei*) involved targeting cytochrome P450, cyclin-dependent kinase 6, and caspases 3 and 8. This was indicated by cell damage observed through TEM.

**Conclusion:**

This study provides comprehensive results on the potential of Majapahit fruit as a natural antibacterial agent. Thus, Majapahit fruit can be considered for functional food applications.

## Introduction

The major problems in vannamei shrimp (*Litopenaeus vannamei*) farming are primarily caused by viral and bacterial diseases such as white spot syndrome virus and vibriosis. These diseases have been widely spread through various ways in the Indonesian shrimp industry [1]. They lead to the death of shrimp seeds or larvae. Infection with pathogenic microorganisms leads to a decrease in the production of many cultivated aquatic organisms and, consequently, high economic losses. Vibriosis (caused by the bacteria in the genus *Vibrio*) has been identified as a major problem for shrimp farmers. *Vibrio* bacteria attack shrimp larvae when the shrimp is in a stressed environment and weak conditions [2].

While antibiotics may be considered a potential solution to this problem, their use in aquaculture environment is expensive, causes the accumulation of several harmful residues in the shrimp/fish, and leads to the rise of antibiotic-resistant pathogens [3]. The bioaccumulation negatively affects consumer health, such as immunosuppression, accumulation of residues in the tissues, and occurrence of drug-resistant pathogens. Further, it causes shrimp/fish mortality and environmental pollution.

The methanol extract of Majapahit fruit (*Crescentia cujete*) has been shown to effectively inhibit the growth of *Vibrio harveyi*, unlike its extracts in other solvents. Phytochemical tests showed that Majapahit fruit contains flavonoids, saponins, and triterpenoids [4]. Majapahit fruit extract has antibacterial properties because its bioactive constituents can inhibit bacterial growth by disrupting cell wall permeability and inactivating protein permeability and transport.

This study aimed to evaluate the antibacterial properties of Majapahit fruit against *V. harveyi* both *in vitro* and *in silico*.

## Materials and Methods

### Ethical approval

This study does not need ethical approval.

### Column chromatography

Column chromatography was performed as described by Maryani [5]. Briefly, isolation was performed after obtaining the best eluent combination and column chromatography was performed by installing the column perpendicularly and glass wool was inserted at the bottom of the column. Before introducing the extract, the column was washed and silica gel solution was poured into the column. A total of 13-15 g of silica gel was dissolved in the eluent to obtain the silica gel solution, and then, the eluent was added again with an eluent ratio of 1: 1 (w/v). The column length used was 60 cm and the length of the silica gel in the column was 30-35 cm.

### Bacterial *V. harveyi* culture

*V. harveyi* was cultured using liquid nutrient broth media and solid thiosulfate-citrate-bile salts-sucrose media. Pure *V. harveyi* were obtained from Brackish Water Aquaculture Development Center (BBAP), Jepara, Indonesia. The bacteria were rejuvenated before conducting the antibacterial tests.

### Determination of minimum inhibitory concentration (MIC) and transmission electron microscopy (TEM)

The antibacterial activity of Majapahit fruit extract was determined using the disc test (6 mm) with various doses *in vitro*. The basic principle of this method is to allow the extract to diffuse into a solid bacterial culture [6-8]. The identification of cell damage caused by *V. harveyi* was visualized through TEM.

### *In silico* analysis

Software and databases such as PubChem^®^ (http://pubchem.ncbi.nlm.nih.gov/), Pass Online (http://www.pharmaexpert.ru/passonline/), Search Tool 17 Interacting Chemicals/STITCH (http://sttitch.embl.de/cgi/show_input_page.pl), and UNIPROT were used. The biological activity of the compounds obtained from gas chromatography-mass spectrometry was analyzed using Pass Online software. The antibacterial mechanism against *Vibrio* spp. was predicted using STITCH and the bacterial protein was identified using UNIPROT.

## Results and Discussion

### Antibacterial activity test

Three different fractions of the fruit extract were obtained through column chromatography, and then, the antibacterial test was performed to determine the best fraction that could inhibit the growth of *V. harveyi*. The fraction that provided the largest inhibition zone was fraction 3 (23.11 mm) followed by fraction 2 (14.16 mm) and fraction 1 (11.58 mm) (Table-1). In this study, the inhibition zone that was produced by fraction 3 was larger than that by methanol extract of Majapahit fruit, which was about 17.29 mm [9]. These findings indicated that the compounds in the Majapahit fruit extract and fraction showed a synergistic effect to inhibit *V. harveyi* growth. Another study about the synergistic effect of the extract and fraction of Kopasanda (*Chromolaena odorata* L.) leaves [8] and *Padina australis* leaves [10] has been performed. The inhibition zone was measured to determine the strength of an antimicrobial agent against the bacteria. The resistance around the disc depends on the absorption capacity of the active compound. If the antimicrobial agent is inhibited, the bacterial growth stops, and the zone around the disc will be visible as a clear circle that is not overgrown with bacteria after incubated for 18-24 h [11].

**Table-1:** Inhibitory Zone Diameter of Majapahit fruit fraction (*Crescentia cujete*) against *V. harveyi*.

Inhibition zone (mm)		
Fraction 1	Fraction 2	Fraction 3
11.58^b^	7.67^c^	23.11^a^

Means with different superscripts are significantly different at p<0.05.

According to Rinawati [12], if the inhibition zone is <10 mm in size, the inhibitory response is categorized as non-existent. The inhibitory response is categorized as weak for an inhibition zone of 10-15 mm in size, average for 16-20 mm, and strong for more than 20 mm. Thus, fraction 3 of Majapahit fruit (inhibition zone of 23.11 mm) had a strong inhibitory response against bacterial growth. Compared with the previous studies with the same bacteria *V. harveyi*, the inhibition zones observed in this study were lower than those observed in the study by Harlina *et al*. [8] who reported an inhibition zone of ~19 mm produced by Kopasanda (*C. odorata* L.) leaf extract. The measurements are higher than those reported for pomegranates (26 mm), apples (20 mm), and lemons (20 mm) against *Staphylococcus aureus* [13]. Meanwhile, the inhibition zones in this study were larger compared to those in the study by Rinawati [12] using wet Majapahit extract on *Vibrio alginolyticus* (by 8.8 mm) and Majapahit leaf extract (12.4 mm) against *Ralstonia solanacearum* bacteria [14]. Similarly, sweet lime and tomato fruit showed an inhibition zone of 10 mm [13] and seaweed extract (*Halimeda opuntia*) showed an inhibition zone of 15.37 mm [15].

Factors that affect the size of the zone of inhibition of bacterial growth include functional group activity and concentration of the antibacterial substance, resistance of bacteria to the substance, and density of bacterial inoculum. The ability of Majapahit fruit extract to inhibit bacterial growth is strongly influenced by the solvent used in the extract. Polar solvents (methanol) give better results compared to semi-polar (ethyl acetate) and non-polar (n-hexane) solvents.

### MIC

The MIC analysis of Majapahit fruit extract (Table-2) showed that its active compounds were capable of inhibiting the growth of *V. harveyi* at various concentrations. Majapahit fruit extract concentrations in the range of 0.313 mg/mL-10 mg/mL completely inhibited the growth of *V. harveyi*, while lower concentrations of 0.078 mg/mL and 0.165 mg/mL showed bacterial growth. Thus, the concentration of 0.313 mg/mL is the minimum concentration with an inhibition zone of 16.79±0.53 mm. Inhibition zone formed by the influence of the extract of Majapahit fruit is influenced by among other types of test bacteria. *V. harveyi* are Gram-negative bacteria that have cell walls with a thin peptidoglycan layer (5-10% of the total cell wall) [16]. The outer membrane is composed of lipopolysaccharide, lipoproteins, phospholipids, and porins [17]. It serves as a semipermeable barrier against antibiotics, digestive enzymes, and drought conditions [18].

**Table-2:** The Inhibition zone of Majapahit (*Crescentia cujete*) fruit extract at various concentrations.

Treatment (mg/mL)	Average±SD	Inhibition Zone		Note

		24 h	48 h	
10	21.48±0.36	Clear	Clear	Bacterisida
5	19.92±0.21	Clear	Clear	Bacterisida
2.5	19.20±0.28	Clear	Clear	Bacterisida
1.25	18.43±0.62	Clear	Clear	Bacterisida
0.625	17.02±0.26	Clear	Clear	Bacterisida
0.313	16.79±0.53	Clear	Strong	Bacteriostatic
0.165	-	-	-	-
0.078	-	-	-	-

Antibacterial compounds generally act by destroying cell walls, changing membrane permeability, disrupting protein synthesis, and inhibiting enzyme action [16]. Several compounds such as phenol, flavonoids, and alkaloids can disrupt the bacterial cell wall by decomposing phospholipids into glycerol, carboxylic acids, and phosphoric acids. Consequently, the membranes become leaky and are unable to preserve the cell shape. The substances then penetrate the cells, disrupt metabolism, and lead to bacterial lysis.

After 24 h incubation with 0.625-10 mg/mL of Majapahit fruit, no bacterial growth was observed (Table-2), indicating that Majapahit fruit extract is bactericidal. However, incubation with 0.313 mg/mL of Majapahit fruit for 24 h produced a clear zone, indicating inhibition of bacterial growth successfully. However, after incubation for 48 h, the inhibition zone was cloudy, indicating bacterial growth. These findings revealed that Majapahit fruit extract is a promising antibacterial agent because it was capable of inhibiting the growth of bacteria.Thus, 0.313 mg/mL is the minimum concentration of Majapahit fruit extract required to inhibit the growth of *V. harveyi*. This suggests that the Majapahit fruit extract has a strong ability to inhibit the growth of *V. harveyi* even in low concentrations. According to Alcaide *et al*. [19], strong active extracts have MIC values <1.6 mg/mL and may be considered to be potential natural antibacterial agents. Thus, Majapahit fruit extract can be developed to be a natural antibacterial agent against *V. harveyi* which causes vibriosis.

### TEM

**Figure-1:**
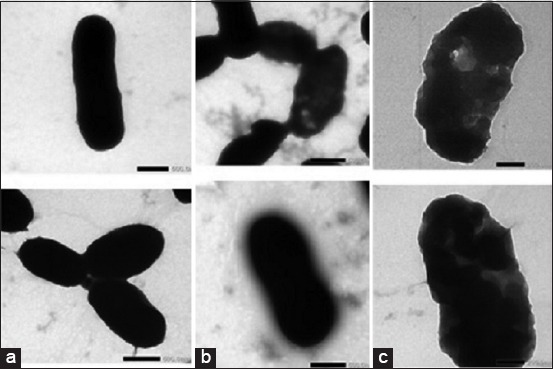
The results of bacterial morphology examination *Vibrio harveyi* using transmission electron microscopy (a) Normal; (b) observation after 3 h; (c) observation after 6 h.

TEM results showed that the bacterial cells were intact without cellular damage in the *V. harveyi* group without Majapahit fruit treatment (Figure-1a). Incubation with Majapahit fruit extract for 3 h showed an increase in cell damage, especially in the cell wall (Figure-1b and c). The active ingredients in the extract of Majapahit fruit react with the components of *V. harveyi* cell wall. Essential oils of *Juniperus rigida* Sieb. leaves extract have been shown to cause the release of the intracellular components of *K. pneumoniae* cells, indicated by a deformed cell morphology and DNA and RNA damage after 4, 8, and 24 h of incubation [20]. During the breakdown of cell membranes, the -OH groups of phenol and flavonoid compounds attack the polar phosphate group into glycerol, carboxylic acid, and phosphoric acid [21]. Consequently, the cell membranes leak, leading to bacterial cell death. Phenolic compounds can disrupt the cell wall and the cytoplasmic membrane, nucleic acid synthesis, and oxygen consumption by affecting the electron transfer chains in pathogenic bacteria [22].

### *In silico* analysis

The biological activity of selected compounds with the probability of the compound to be active (P_a_) >0.3% (P_a_% value close to 1) indicated a trend of increase in activity. Analysis of biological activity and target compounds based on Pass Online and Swiss Target Prediction (http://www.swisstargetprediction.ch/) (Table-3) showed that there were only three biological activities (red colored in the table) that matched the targets of the selected compounds: (1) Cytochrome P450 targeted by furfural/furancarboxaldehyde (0.66%), 4H-pyran-4-one (0.680%), 2-propenoic acid, 3-phenyl (0.41%), 1.2.3-benzenetriol (0.55%), and quercetin (0.10% and 0.34%); (2) CYP1A2 substrate targeted by 4H-pyran-4-one (0.37%), 2-propenoic acid, 3-phenyl (0.49%), 1.2.3-benzenetriol (0.50%), and quercetin compounds (1.00% and 0.91%); and (3) cyclin-dependent kinase 6 inhibitors targeted by 1.2.3-benzenetriol (0.14%) and quercetin compounds (1.0% and 0.37%). There was some biological activity of the selected compounds on other targets, namely, CYP1A substrate, CYP1A inducer, CYP1B1 inhibitor, CYP2J substrate, CYP2A6 substrate, CYP2J substrate, CYP2C8 inhibitor, CYP2C substrate, CYP3A4 inducer, CYP3A inducer, caspase 8 stimulant, and caspase 3 stimulant.

**Table-3:** Biological activity and target compounds.

No.	Biological activity/target compound	Pa value (%)											

		1		2		3		4		5		6	
					
		PS	SW	PS	SW	PS	SW	PS	SW	PS	SW	PS	SW
1	CYP1A2 substrate	-	-	-	-	-	0.38	-	0.49	-	0.51	1.0	0.91
2	Cytochrome P450 stimulant	-	0.66	-	-	-	0.68	-	0.41	-	0.55	1.0	0.34
3	Cyclin-dependent kinase 6 inhibitor	-	-	-	-	-	-	-	-	-	0.14	1.0	0.37
4	CYP1A substrate	-	0.30	-	-	-	0.36	-	0.47	-	0.54	-	0.95
5	CYP1A1 inducer	-	-	-	-	-	-	-	0.46	-	0.53	-	0.94
6	CYP1B1 inhibitor	-	-	-	-	-	-	-	-	-	0.38	-	0.88
7	CYP2J substrate	-	0.74	-	0.59	-	0.84	-	0.90	-	0.83	-	0.68
8	CYP2A6 substrate	-	0.39	-	-	-	0.55	-	0.72	-	0.54	-	0.59
9	CYP2J substrate	-	0.74	-	0.59	-	0.84	-	0.90	-	0.83	-	0.68
10	CYP2C8 inhibitor	-	-	-	0.48	-	-	-	0.67	-	0.67	-	0.77
11	CYP2C substrate	-	-	-	-	-	-	-	0.58	-	0.50	-	0.74
12	CYP3A4 inducer	-	0.43	-	-	-	0.54	-	-	-	0.56	-	0.83
13	CYP3A inducer	-	0.42	-	-	-	0.52	-	-	-	0.55	-	0.79
14	Caspase 8 stimulant	-	0.39	-	-	-	0.57	-	0.44	-	0.55	-	0.43
15	Caspase 3 stimulant	-	0.34	-	-	-	0.51	-	0.50	-	0.56	-	0.50

PS = Pass Online; SW= Swiss Target

Compound Name: (1) Furfural / Furancarboxaldehyde, (2) Pyrazole, 1,4-dimethyl, (3) 4H-Pyran-4-one, (4) 2-propenoic acid, 3-phenyl, (5) 1.2.3-Benzenetriol, (6) Quercetin

### Pathway mechanism

**Figure-2:**
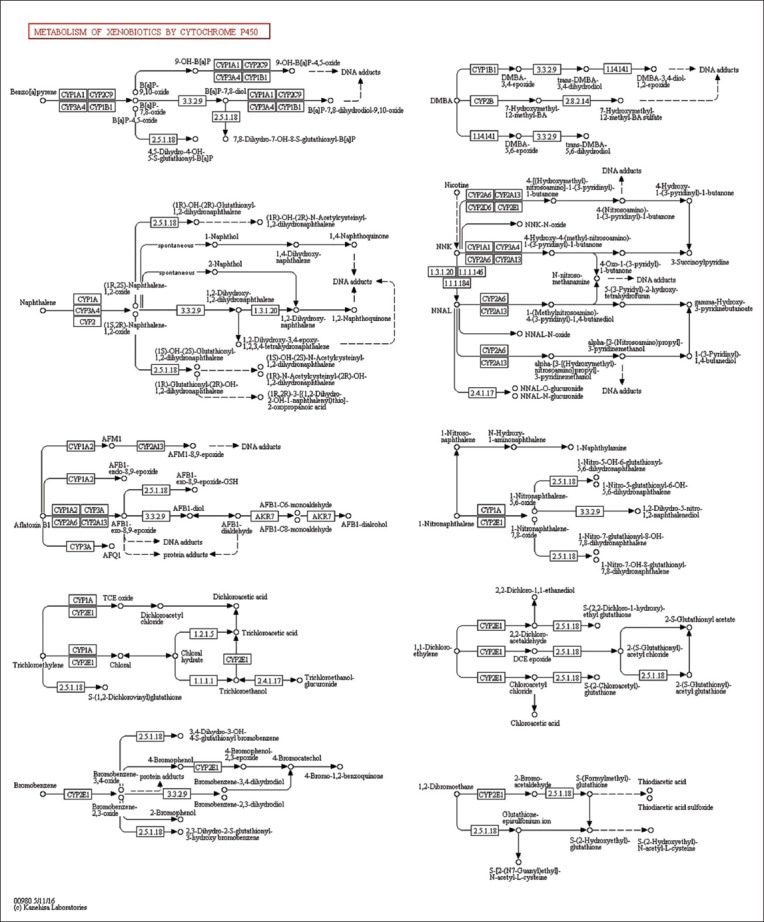
Pathway mechanism of xenobiotic metabolism by cytochrome P450. Furfural/furancarboxaldehyde, 2-propenoic acid, 3-phenyl, pyrazole, 1,4-dimethyl, 1.2.3-benzenetriol, 4H-pyran-4-one, and quercetin are the path of the target compound.

The results showed that there were four pathways through which the selected compounds (furfural/furancarboxaldehyde, 2-propenoic acid, 3-phenyl, pyrazole, 1,4-dimethyl, 1.2.3-benzenetriol, 4H-pyran-4-one, and quercetin) acted on the target compounds (Figure-2):

1. Benzo[a]pyrene pathway, with the target compounds CYP1A1, CYP2C9, CYP3A4, and CYP1B1 causes DNA damage
2. Naphthalene pathway, with the target compounds CYP1A, CYP3A4, CYP2A6, CYP1A1, CYP3A4, and CYP2A6 also causes damage to the DNA
3. Aflatoxin pathway, with the target compounds CYP1A2, CYP2A6, CYP3A, and CYP1A causes DNA and protein damage
4. Trichloromethylene pathway, with only one target compound, CYP1A.

The previous studies have suggested that ring B of flavonoid plays a role in hydrogen bonding with the accumulation of nucleic acid bases and this may explain the inhibition of DNA and RNA synthesis. Chalcones, both fluorinated and chlorinated in position 4 of ring B, are known to have antibacterial potency [23].

**Figure-3:**
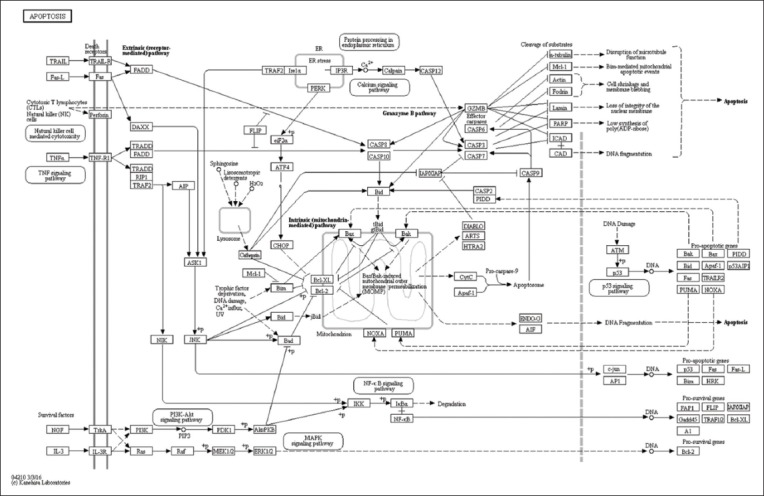
Pathway mechanism of apoptosis. Furfural/furancarboxaldehyde, 2-propenoic acid, 3-phenyl, 1.2.3-benzenetriol, 4H-pyran-4-one, and quercetin with target compounds caspase 8 and caspase 3 stimulant.

In the pathway mechanism in Figure-3, the targets of furfural/furancarboxaldehyde, 2-propenoic acid, 3-phenyl, 1.2.3-benzenetriol, 4H-pyran-4-one, and quercetin are caspases 3 and 8. When cells recognize a virus or a genetic mutation, they may undergo apoptosis (programmed cell death) to prevent cell damage caused by the spread of pathogens. Apoptosis occurs when the cell is under stress, such as oxidative or radiation-based stress. Apoptosis, which is stimulated by caspases, causes the breakdown of cellular components by upregulating the expression of caspase-activated DNAse for DNA fragmentation.

**Figure-4:**
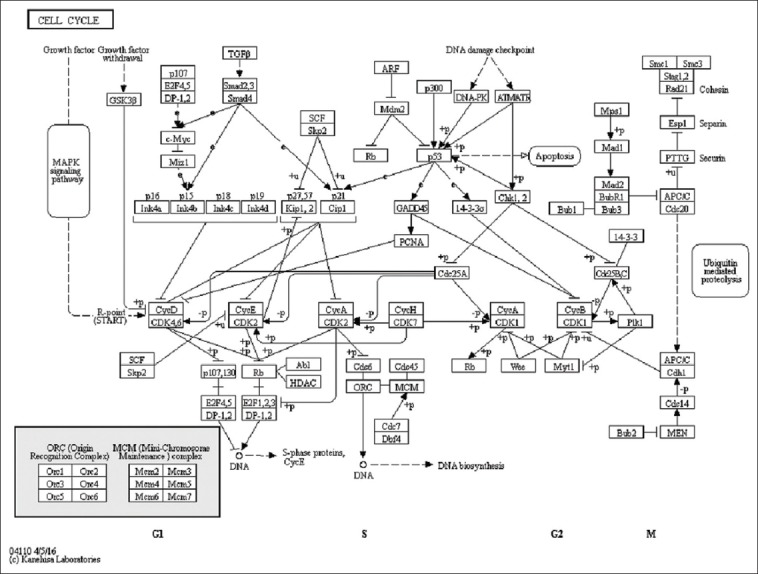
Pathway mechanism of the cell cycle. The target compound of quercetin is cyclin-dependent kinase 6 inhibitor.

Based on the analysis of the cell cycle pathway mechanism (Figure-4), one of the compounds that play a role in the antibacterial action is quercetin. The previous studies have reported significant inhibition of bacterial motility by quercetin. Quercetin also acts as a bacteriostatic compound because it can inhibit ligation of D-Ala-D-Ala in bacterial cells, by inhibiting D-alanine: D-alanine ligase and preventing bacterial growth [24,25]. Thus, quercetin is a well-suited compound for antibacterial drugs.

## Conclusion

The results showed that the MIC of Majapahit fruit extract against *V. harveyi* was 0.313 mg/mL. The pathogenic mechanism of *V. harveyi* on vannamei shrimp works on the target compounds of cytochrome P450, cyclin-dependent kinase 6, and caspases 3 and 8. This is indicated by cell damage observed through both histological examinations and TEM.

## Authors’ Contributions

SR designed the research and wrote the manuscript. HP reviewed the manuscript. Both authors read and approved the final manuscript.
